# High-intensity interval training and moderate-intensity continuous training in adults with Crohn’s disease: a pilot randomised controlled trial

**DOI:** 10.1186/s12876-019-0936-x

**Published:** 2019-01-29

**Authors:** Garry A. Tew, Dean Leighton, Roger Carpenter, Simon Anderson, Louise Langmead, John Ramage, James Faulkner, Elizabeth Coleman, Caroline Fairhurst, Michael Seed, Lindsay Bottoms

**Affiliations:** 1 Department of Sport, Exercise and Rehabilitation, Northumbria University, Newcastle-upon-Tyne, NE1 8ST UK; 2 Centre for Immunobiology, Queen Mary University of London, Blizard Building, Newark Street, Whitechapel, London, E1 2AT UK; 3 School of Health, Sport and Bioscience, University of East London, Stratford Campus, London, E15 4LZ UK; 4 Guy’s and St Thomas’ NHS Foundation Trust, London, UK; 5 Digestive Diseases Clinical Academic Unit, Barts and the London NHS Trust, London, UK; 6 Hampshire Hospitals NHS Foundation Trust, Hampshire, UK; 7 Department of Sport, Exercise and Health, University of Winchester, Winchester, SO22 4NR UK; 8 York Trials Unit, University of York, Heslington, York, YO10 5DD UK; 9 Centre for Psychology and Sports Science, University of Hertfordshire, Life and Medical Sciences, CP Snow Building, College Lane, Hatfield, AL10 9AB UK

**Keywords:** Inflammatory bowel disease, Exercise therapy, Randomised controlled trial

## Abstract

**Background:**

This study assessed the feasibility and acceptability of two common types of exercise training—high-intensity interval training (HIIT) and moderate-intensity continuous training (MICT)—in adults with Crohn’s disease (CD).

**Methods:**

In this mixed-methods pilot trial, participants with quiescent or mildly-active CD were randomly assigned 1:1:1 to HIIT, MICT or usual care control, and followed up for 6 months. The HIIT and MICT groups were offered three exercise sessions per week for the first 12 weeks. Feasibility outcomes included rates of recruitment, retention, outcome completion, and exercise attendance. Data were collected on cardiorespiratory fitness (e.g., peak oxygen uptake), disease activity, fatigue, quality of life, adverse events, and intervention acceptability (via interviews).

**Results:**

Over 17 months, 53 patients were assessed for eligibility and 36 (68%) were randomised (47% male; mean age 36.9 [SD 11.2] years); 13 to HIIT, 12 to MICT, and 11 to control. The exercise session attendance rate was 62% for HIIT (288/465) and 75% for MICT (320/429), with 62% of HIIT participants (8/13) and 67% of MICT participants (8/12) completing at least 24 of 36 sessions. One participant was lost to follow-up. Outcome completion rates ranged from 89 to 97%. The mean increase in peak oxygen uptake, relative to control, was greater following HIIT than MICT (2.4 vs. 0.7 mL/kg/min). There were three non-serious exercise-related adverse events, and two exercise participants experienced disease relapse during follow-up.

**Conclusions:**

The findings support the feasibility and acceptability of the exercise programmes and trial procedures. A definitive trial is warranted. Physical exercise remains a potentially useful adjunct therapy in CD. [ID: ISRCTN13021107].

**Electronic supplementary material:**

The online version of this article (10.1186/s12876-019-0936-x) contains supplementary material, which is available to authorized users.

## Background

Regular exercise training has been recommended as an adjunct therapy for people with Crohn’s disease (CD) [1–3] because of its potential beneficial effects on physical fitness, mental health, and disease-related factors such as fatigue, bone mineral loss and inflammation [4–6]. However, empirical evidence on the effects of exercise training in CD is sparse, with only a handful of intervention studies [7–11], some of which have methodological limitations, such as short follow-up, no control group, and a small sample size. Among adults with other chronic inflammatory diseases, a traditional model of exercise prescription has been moderate-intensity continuous training (MICT; e.g. 30–60 min of moderate-intensity continuous endurance-type exercise such as swimming, cycling or running performed 3–5 times per week) [12], but a growing body of evidence indicates that high-intensity interval training (HIIT; e.g. 0.5–4 min bouts of vigorous exercise interspersed by periods of passive or active recovery) is a more time-efficient exercise strategy, eliciting similar or even superior cardio-metabolic adaptations compared to MICT, at least when compared on a work-matched basis [13–16]. There has only been one published study investigating HIIT in CD patients to date [17], which showed that a single session of cycle-based HIIT was well tolerated and did not markedly increase pro-inflammatory cytokines (e.g., TNF-α) in a group of 15 teenagers. A greater understanding of the feasibility, acceptability and effects of different types of exercise training is needed to support the development evidence-based exercise guidelines and promotion strategies that are specific to CD.

We hypothesised that supervised endurance exercise training (either as HIIT or MICT) is a safe and effective strategy for improving cardiorespiratory fitness, fatigue, quality of life and mental health in people with CD. Before embarking on a full-scale randomised controlled trial to test this hypothesis, we conducted a pilot trial to address several areas of uncertainty. For example, the possibility that many potential participants would be of working age and have disease-specific barriers to exercise (e.g., fatigue [18]) raised questions about the ability to recruit and retain individuals with CD to a clinical trial of supervised exercise training. Hence, the main aims of the Exercise for Adults with Crohn’s disease Trial (EXACT) study were to determine the acceptability and potential benefits and harms of HIIT and MICT in adults with quiescent or mildly-active CD, and the feasibility of conducting a full-scale trial.

## Methods

### Study design and setting

A full description of the methods has been published [19]. The study was a multi-centre, three-arm, parallel-group, pilot randomised controlled trial. Participants were randomised 1:1:1 to receive usual care, usual care plus HIIT or usual care plus MICT. Study assessments were conducted at baseline and at 3 and 6 months after randomisation. Recruitment was from three hospital trusts in England: Guy’s and St Thomas’ NHS Foundation Trust, Barts Health NHS Trust, and Hampshire Hospitals NHS Foundation Trust. The exercise programmes were delivered in the exercise science facilities of the University of East London and the University of Winchester. Data management and statistical analysis took place at York Trials Unit, University of York. Ethics approval was granted by the Camden and Kings Cross Research Ethics Committee (reference 15/LO/1804), and all participants provided written informed consent before enrolment. The trial was registered prospectively (ISRCTN13021107).

### Participants

We included male and female patients between 16 and 65 years of age with a clinical diagnosis of CD. Patients had to have a stool calprotectin of < 250 μg/g, stable medication (> 4 weeks), and quiescent or mildly-active disease, as indicated by a Crohn’s Disease Activity Index (CDAI) of < 150 or 150–219, respectively. Exclusion criteria were: contraindication to exercise testing or training [20], coexistent serious autoimmune disease (e.g. rheumatoid arthritis or systemic sclerosis), pregnant, planned pregnancy or major surgery within the first 3 months after randomisation, poor tolerability of venepuncture or inadequate access for venous blood sampling, and current participation in > 90 min/week of purposeful exercise (e.g. cycling, swimming or running) or another clinical trial.

### Randomisation and allocation concealment

A statistician at York Trials Unit managed the randomisation process. Following baseline assessment, a research assistant emailed the statistician for notification of the participant’s group allocation. Participants were randomly assigned 1:1:1 to one of the three study groups using a computer-generated randomisation schedule stratified by centre and baseline disease status (inactive [CDAI < 150] or mild [CDAI 150–219]) using randomly permuted blocks of sizes 3 and 6. The block sizes and allocation sequence were not disclosed to ensure concealment.

### Interventions

All three groups received usual care, which comprised evidence-based medical treatment optimisation. Participants allocated to usual care did not receive any supervised exercise or exercise advice as part of the trial; however, following the final study assessment they were offered a telephone-based consultation with a research assistant who discussed their individual facilitators/barriers to exercise, and provided guidance on incorporating physical activity into their lifestyle.

Participants allocated to the HIIT and MICT groups were invited to complete three supervised exercise sessions per week for 12 consecutive weeks, commencing the week following their baseline assessment and randomisation. Reimbursement was provided for travel expenses. All exercise was undertaken on a leg cycle ergometer (Lode Corival or SRM Ergometer), with each session comprising a 5-min warm-up at 15% of peak power output (Wpeak; determined during the baseline cardiopulmonary exercise test), a main conditioning phase, and then a 3-min cool-down at 15% Wpeak. For HIIT, the conditioning phase involved ten 1-min bouts at 90% Wpeak, interspersed with 1-min bouts at 15% Wpeak (total session duration = 28 min), whereas for MICT it involved 30 min at 35% Wpeak (total session duration = 38 min). Heart rate (Polar FT1, Polar Electro, Kempele, Finland), differential ratings for central (i.e. cardiopulmonary sensations) and leg exertion (RPE-C and RPE-L, respectively; Borg CR-10 scale [21]), and general affective valence (i.e. pleasure and displeasure; 11-point feeling scale) were recorded at regular intervals during each session. The feeling scale data will be published elsewhere. Incremental cycle exercise testing to maximum volitional exertion was performed in the final sessions of weeks 4 and 8 to re-calculate Wpeak and determine if the power output of the upcoming exercise sessions needed to be changed.

After the initial 12-week supervised training period, all exercise group participants were encouraged to continue a similar exercise regime in their own home or community setting without the support of the trial team.

### Feasibility and acceptability outcomes

Trial feasibility outcomes included rates of recruitment, retention, and outcome completion. Barriers and facilitators to recruitment were also identified using a standardised questionnaire [22], which was completed by trial staff who had a responsibility for recruitment. The acceptability of the exercise programmes was assessed using group preference data (assessed before randomisation), rates of session attendance and completion, a measure of exercise enjoyment completed at 3 months after randomisation (Physical Activity Enjoyment Scale, PACES [23]), and participant feedback via telephone interviews conducted after the 6-month assessments. The safety of the exercise programmes was also assessed by exploring rates of disease relapse at 3 months, the number and type of adverse events, drop-out rates, and reasons for withdrawal in each group. Relapse was defined as an increase in CDAI of ≥100 points to a score ≥ 150 [24].

We pre-specified that this pilot trial would be deemed successful and lead to the development of a proposal for a full-scale trial if: (i) at least one of the exercise programmes was shown to be acceptable, based principally on participant feedback (i.e. interview data) and exercise session attendance data (acceptable attendance defined as at least 67% of participants completing at least 24 of the 36 sessions); (ii) at least 24 patients being recruited within 12 months, and; (iii) complete data on cardiorespiratory fitness, CDAI, and quality of life at 3 months for at least 67% of participants.

### Behaviour, fitness and health outcomes

The following outcome measures were assessed in all participants at baseline and 3 months after randomisation: body mass, stature, waist circumference, blood pressure, resting heart rate, cardiorespiratory fitness (ventilatory threshold and peak oxygen uptake recorded during incremental cycle ergometer testing to maximum volitional exertion), disease status (CDAI), intestinal inflammation (faecal calprotectin), and blood markers of inflammation (T lymphocyte subsets [Th1/Th2/Th17] and various cytokines including IL-6, IL-10, TNF-α and C-reactive protein; data to be published elsewhere). Standard questionnaires were also administered at baseline and 3 and 6 months after randomisation, including the Inflammatory Bowel Disease Quality of Life Questionnaire (IBDQ [25]), EuroQol EQ-5D-5 L (to measure health-related quality of life, [26]), IBD Fatigue Scale [27], Hospital and Anxiety Depression Scale (HADS [28]), and International Physical Activity Questionnaire-long (IPAQ [29]). (Please note that the published trial protocol contains a typographical error in that it states that the short version of the IPAQ would be administered).

### Sample size

Following sample size guidelines for pilot studies [30], we aimed to have at least 12 participants in each group complete the study. To allow for up to 20% attrition, an overall target of 45 participants was used (15 per group).

### Blinding

Due to the nature of the trial, blinding of participants and intervention facilitators to group allocation was not possible. Questionnaires were completed by participants independently and checked by a researcher for completeness. Anthropometric, cardiorespiratory fitness and disease activity outcomes were assessed by researchers blinded to group allocation. Participants were asked not to disclose their allocation.

### Statistical analysis

**Fig. 1:**
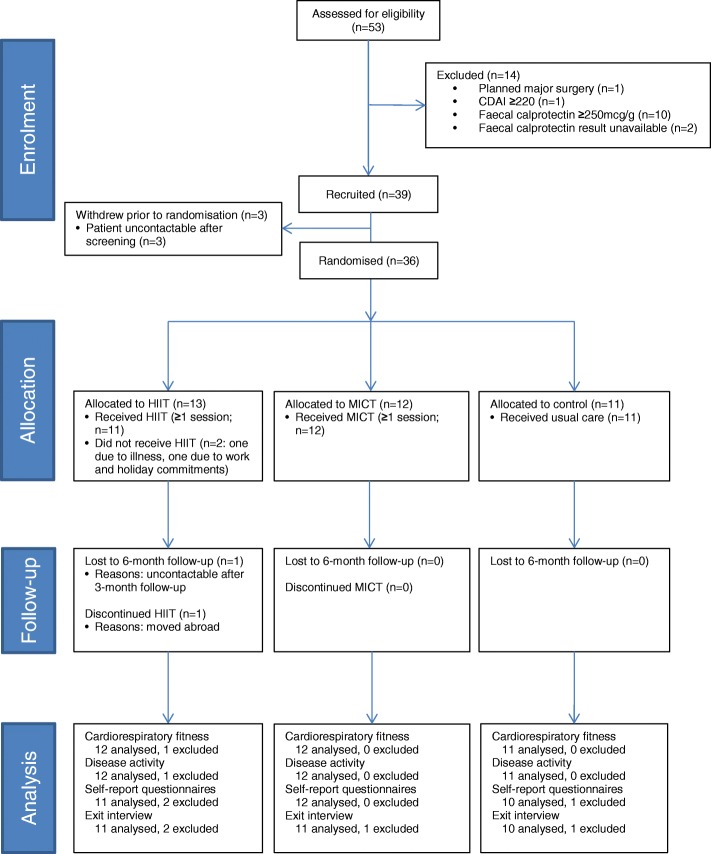
Flow of participants through the trial

Data from paper case report forms were entered and checked for missing and invalid values in Microsoft Excel® then imported into Stata v15 (StataCorp) for analysis. The flow of participants through the trial is presented in a CONSORT diagram (Fig. 1). Baseline data are summarised descriptively by trial arm. The guidance around analysing pilot studies states that no formal hypothesis testing should be undertaken [31], and as such quantitative outcome data are summarised using descriptive statistics only, using the principles of intention to treat. Exit interviews were analysed using qualitative content analysis [32].

## Results

Recruitment took place between May 2016 and September 2017, with all follow-up data collection completed by March 2018. The trial was stopped at the end of the grant funding interval, with the minimum recruitment target having been achieved.

### Recruitment, group allocation and participant characteristics

Of 53 patients who were fully assessed for eligibility, 39 met all eligibility criteria and 36 were randomised (Fig. 1). A median of 2 participants were recruited per month (range 1 to 6). The three sites recruited 19, 12 and five participants each. The most common reason for exclusion was having active disease (*n* = 11). Interview data (analysed for *n* = 31; summarised in Additional file 1) revealed that most participants were recruited via face-to-face approach in clinic, with the most common reasons for enrolment being potential health benefits (*n* = 20), altruistic motives (*n* = 12), and it being seen as a good way to start an exercise regime (*n* = 10). Five investigators provided feedback on barriers and facilitators to recruitment (Additional file 2). These investigators and several participants suggested that recruitment might have been easier had there been more exercise venues. An investigator from the site with the least participants (Hampshire Hospitals NHS Foundation Trust; *n* = 5), also stated that their recruitment was hampered by there being fewer eligible patients than expected; most patients attending their clinics had active disease.

Thirteen participants were allocated to HIIT, 12 to MICT, and 11 to control (Fig. 1). Of the 27 participants who expressed a preference for a specific group before allocation, 20 (74%) preferred HIIT, 6 (22%) MICT, and one (4%) control. Interviewees recognised the need for a control group, and although control participants were generally disappointed with their allocation, they were willing to complete the study. All but one control participant reported maintaining their pre-trial exercise habits during the follow-up period, with the remaining participant explaining that they had started doing aerobic and strength training 3–4 times per week shortly after randomisation. One exercise participant said that they would have dropped out if they had been allocated to control.

Table 1 shows the participant characteristics at baseline. The groups appear well balanced for the majority of variables. Seventeen participants (47%) were male and the mean age was 36.9 years (SD 11.2). A higher proportion of participants were male in the HIIT (54%) and control (64%) groups than the MICT group (25%). Most participants were of white ethnicity (78%), had quiescent disease (89%), and were in paid employment (78%). The mean time since diagnosis was 13.7 years, ranging from 4 months to 38.2 years. Twenty-six participants (72%) reported having slight-to-moderate fatigue and eight (22%) reported having severe fatigue. There were very few comorbidities, which included asthma (*n* = 3), anaemia (*n* = 2), diabetes (*n* = 1), ankylosing spondylitis (*n* = 1), and bipolar disorder (*n* = 1). The most common medication used for CD was immunosuppressants (47%) and biologics (33%). The most common previous surgery for CD was right hemicolectomy (*n* = 11).

**Table 1:** Baseline demographics and clinical characteristics

	HIIT (*n* = 13)	MICT (*n* = 12)	Control (*n* = 11)	All (*n* = 36)
Age, years	37.0 (11.1)	38.5 (13.0)	35.0 (10.0)	36.9 (11.2)
Male sex, n (%)	7 (54)	3 (25)	7 (64)	17 (47)
White ethnicity, n (%)	10 (77)	11 (92)	7 (64)	28 (78)
Body mass, kg	76.2 (13.5)	63.8 (12.5)	69.8 (13.2)	70.1 (13.8)
Current smoker, n (%)	1 (8)	1 (8)	2 (18)	4 (11)
Employment status, n (%)				
Working full- or part-time	9 (69)	10 (83)	9 (82)	28 (78)
Student	1 (8)	1 (8)	2 (18)	4 (11)
Other	3 (23)	1 (8)	0 (0)	4 (11)
CD duration, years	16.1 (11.9)	11.5 (10.9)	13.7 (9.8)	13.7 (10.8)
CD location, n (%)				
Ileum	4 (31)	3 (25)	4 (36)	11 (31)
Colon	3 (23)	4 (33)	1 (9)	8 (22)
Ileum and colon	6 (46)	4 (33)	5 (46)	15 (42)
Missing	0 (0)	1 (8)	1 (9)	2 (6)
CD activity status, n (%)				
Inactive	11 (85)	11 (92)	10 (91)	32 (89)
Mildly active	2 (15)	1 (8)	1 (9)	4 (11)
CDAI	74 (48)	55 (47)	73 (45)	67 (46)
Faecal calprotectin, μg/g	89 (72)	45 (40)	56 (55)	65 (59)
Medication for CD, n (%)				
Immunosuppressants	8 (62)	5 (42)	4 (36)	17 (47)
Biologics	8 (62)	2 (17)	2 (18)	12 (33)
Oral 5-aminosalicylates	0 (0)	3 (25)	2 (18)	5 (14)
Analgesics	3 (23)	0 (0)	1 (9)	4 (11)
Antibiotics	1 (8)	0 (0)	1 (9)	2 (6)
Antidiarrheals	1 (8)	1 (8)	1 (9)	3 (8)
Previous surgery for CD, n (%)				
Defunctioning ileostomy	1 (8)	0 (0)	1 (9)	2 (6)
Drainage of abscess	1 (8)	1 (8)	0 (0)	2 (6)
Excision of fistula	1 (8)	0 (0)	1 (9)	2 (6)
Right hemicolectomy	6 (46)	2 (17)	3 (27)	11 (31)
Small bowel resection	2 (15)	0 (0)	2 (18)	4 (11)
Left hemicolectomy	0 (0)	0 (0)	1 (9)	1 (3)
Panproctocolectomy and	1 (8)	0 (0)	1 (9)	2 (6)
ileostomy				
Perianal surgery	1 (8)	0 (0)	1 (9)	2 (6)
Subtotal colectomy and primary anastomosis	0 (0)	0 (0)	1 (9)	1 (3)
Other	2 (15)	0 (0)	3 (27)	5 (14)

Data are presented as mean (SD) unless otherwise stated

### Trial retention and assessment completion rates

No participants formally withdrew from the study, but one HIIT participant was lost to the 6-month follow-up (Fig. 1). At 3 months (i.e., intervention end-point), 34 (94%) participants completed the hospital visit, and 35 (97%) completed the university visit. At 6 months, 33 (92%) participants completed the postal questionnaire, and 32 (89%) completed the telephone interview. The interviewees stated that the logistics and content of the assessment visits were acceptable (Additional file 1).

### Exercise adherence, enjoyment and acceptability

Of the 465 and 429 exercise sessions that were offered to HIIT and MICT participants, respectively, 288 (62%) and 320 (75%) were attended, giving a combined attendance rate of 68% (608/891). All 608 attended sessions were completed as planned. The mean (SD) power output used in the exercise sessions of weeks 1–4 and 9–12 were 148 (25) W and 173 (36) W respectively for the HIIT group and 50 (14) W and 54 (13) W respectively for the MICT group. The median (range) number of sessions attended was 25 (0–36) and 25 (18–34) for the HIIT and MICT groups, respectively. Eight (62%) of the HIIT participants and eight (67%) of the MICT participants achieved the pre-specified attendance criterion of at least 24 sessions. Two HIIT participants did not attend a single exercise session: one due to illness, and the other due to work and holiday commitments. Another HIIT participant withdrew from the intervention after completing 5 sessions due to moving abroad. The main reasons for sessions being missed were work commitments (25%, 72/286), illness (25%, 71/286 [only two of which were CD-related]) and holiday (14%, 40/286) (data from both exercise groups combined).

The interviews indicated mixed views about there being three sessions per week. Some participants (*n* = 12) stated that this frequency was, or would have been (for controls), difficult to adhere to, whereas others felt this frequency to be achievable (*n* = 11) and necessary for improving fitness (*n* = 6). Two participants indicated that they would not have achieved this frequency had the session times not been as flexible. Three other participants stated that the frequency would have been more achievable had weekend sessions also been offered.

The intensity of training completed by the HIIT participants, based on data recorded at exercise interval 9 of 10, is summarised as follows: mean (SD) RPE-C = 5.1 (1.7) (i.e. ‘hard’), RPE-L = 5.5 (1.6) (i.e. ‘hard’) and heart rate = 92% of maximum (5%). Corresponding values for the MICT participants were: RPE-C = 2.9 (1.5) (i.e. ‘moderate’), RPE-L = 3.3 (1.5) (i.e. ‘moderate’) and heart rate 68% of maximum (6%). None of the interviewees thought that either training programme was too hard or too easy. Two participants were initially concerned that the HIIT might be too hard, but found that this did not turn out to be the case.

All interviewees found cycling to be an acceptable mode of exercise, with some recognising that it could be carefully controlled and was suitable for a range of fitness levels. However, two participants said that the seat was uncomfortable. Six participants stated that they would have also liked to try other exercise modes including muscle-strengthening exercises (*n* = 4), running (*n* = 1), and arm-cranking (*n* = 1). Another six participants stated that they were glad running was excluded, with two participants explaining that it has previously caused them to experience bowel urgency. Feedback on other aspects of the exercise programmes (e.g. duration, setting, provider) is summarised in Additional file 1. The mean (SD) PACES score at 3 months (i.e. intervention end-point) out of a possible 126 was 99.4 (12.9) for HIIT and 101.3 (17.4) for MICT, equating to participants reporting the exercise sessions as ‘enjoyable’.

### Behaviour, fitness and health measures

Summary data for the behaviour, fitness and health measures are presented in Tables 2 and 3. The mean change in peak oxygen uptake from baseline to 3 months, relative to control, was greater following HIIT than MICT (+ 2.4 vs. + 0.7 mL/kg/min). This corresponded with the mean (SD) change in peak power output from baseline to 3 months, which was + 24 W (17) for HIIT, + 12 W (16) for MICT, and + 4 W (14) for control.

**Table 2:** Fitness and health measures at baseline and follow-up

	HIIT	MICT	Control	All
Body mass, kg				
Baseline	76.2 (13.5)	63.8 (12.5)	69.8 (13.2)	70.1 (13.8)
3 months	76.4 (14.4)	63.0 (12.7)	71.0 (13.5)	70.1 (14.3)
Waist circumference, cm				
Baseline	87.3 (11.8)	79.5 (14.7)	83.1 (9.8)	83.4 (12.4)
3 months	86.5 (9.8)	76.8 (12.8)	84.8 (8.5)	82.6 (11.1)
Resting heart rate, beats/min				
Baseline	72 (8)	75 (12)	73 (11)	73 (10)
3 months	72 (10)	74 (9)	76 (10)	74 (10)
Systolic blood pressure, mmHg				
Baseline	130 (12)	122 (17)	126 (13)	126 (14)
3 months	126 (11)	120 (16)	128 (15)	125 (14)
Diastolic blood pressure, mmHg				
Baseline	82 (10)	76 (9)	78 (8)	79 (9)
3 months	78 (9)	74 (10)	79 (10)	77 (9)
Ventilatory threshold, mL/kg/min				
Baseline	16.5 (4.9)	16.0 (4.1)	16.6 (6.1)	16.4 (4.9)
3 months	16.8 (5.5)	18.2 (3.7)	16.1 (4.7)	17.0 (4.7)
Peak oxygen uptake, mL/kg/min				
Baseline	27.3 (7.7)	28.7 (8.6)	28.6 (10.0)	28.2 (8.6)
3 months	29.7 (8.2)	29.3 (6.6)	28.5 (9.2)	29.2 (7.9)
CDAI				
Baseline	74 (48)	55 (47)	73 (45)	67 (46)
3 months	59 (74)	78 (48)	99 (50)	77 (59)
Faecal calprotectin, μg/g				
Baseline	89 (72)	45 (40)	56 (55)	65 (59)
3 months	100 (113)	63 (113)	69 (146)	77 (121)

Data are presented as mean (SD)

**Table 3:** Questionnaire data at baseline and follow-up

	HIIT	MICT	Control	All
IBDQ (32 to 224)^a^				
Baseline	184 (16)	181 (23)	164 (17)	177 (20)
3 months	186 (19)	192 (18)	174 (21)	184 (20)
6 months	180 (20)	189 (22)	175 (23)	182 (22)
EQ-5D (−0.285 to 1)^a^				
Baseline	0.85 (0.13)	0.83 (0.12)	0.70 (0.20)	0.80 (0.16)
3 months	0.85 (0.10)	0.87 (0.13)	0.78 (0.17)	0.83 (0.14)
6 months	0.85 (0.12)	0.83 (0.12)	0.77 (0.22)	0.81 (0.16)
IBD-F, Fatigue (0 to 20)^b^				
Baseline	8.2 (3.0)	7.8 (5.3)	9.3 (4.1)	8.4 (4.1)
3 months	8.3 (3.2)	8.3 (4.9)	7.8 (4.2)	8.1 (4.0)
6 months	7.5 (2.5)	7.3 (4.2)	7.5 (4.0)	7.4 (3.6)
IBD-F, Activities (0 to 120)^b^				
Baseline	22.3 (19.0)	22.7 (22.5)	34.3 (20.5)	26.1 (20.8)
3 months	26.4 (20.5)	25.4 (28.1)	35.0 (20.4)	28.7 (23.1)
6 months	27.7 (12.4)	26.2 (20.6)	32.4 (21.3)	28.7 (18.4)
HADS, Anxiety (0 to 21)^b^				
Baseline	5.5 (3.9)	6.8 (5.2)	7.7 (4.3)	6.6 (4.4)
3 months	5.2 (2.5)	5.5 (3.6)	6.2 (4.2)	5.6 (3.4)
6 months	3.8 (3.5)	5.3 (4.3)	5.5 (3.6)	4.9 (3.7)
HADS, Depression (0 to 21)^b^				
Baseline	3.6 (3.1)	3.8 (2.9)	5.2 (2.9)	4.1 (3.0)
3 months	2.7 (1.7)	2.7 (3.3)	2.6 (2.5)	2.7 (2.5)
6 months	2.7 (1.5)	3.1 (3.1)	4.4 (4.0)	3.4 (3.1)
IPAQ, Total physical activity, MET-min/week, median (IQR)				
Baseline	2874 (1273, 6474)	3237 (1383, 5442)	1602 (526, 2781)	2484 (1028, 4409)
3 months	3618 (1692, 5271)	2099 (1441, 3729)	2928 (2118, 5351)	2897 (1645, 5213)
6 months	1188 (99, 4149)	2163 (1328, 7993)	2817 (2243, 3969)	2557 (1109, 4451)

Data are presented as mean (SD) unless otherwise stated

^a^Higher score is better

^b^Lower score is better

The interviewees reported a range of physical benefits from participating in the exercise programmes, including feeling fitter (*n* = 8) and more energised (*n* = 8), and having a thinner waist (*n* = 1) and more-defined thigh muscles (*n* = 2). Five participants also reported disease-specific benefits, such as reduced inflammation (*n* = 1; based on routine colonoscopy findings), less frequent bowel movements (*n* = 1), and a “calmer gut” (*n* = 1). Mental benefits were less frequently cited, but included generally feeling better (*n* = 2), and improvements in wellbeing (*n* = 3) and mood (*n* = 2). Eight participants said that the study had increased their motivation to exercise in the future, and 12 participants said that they had continued exercising (a variety of regimes) since finishing the supervised sessions.

### Disease activity and safety

Summary data for disease activity (CDAI and faecal calprotectin) are presented in Table 2.

Two participants, one from each exercise group, experienced disease relapse between baseline and 3 months. The HIIT participant was a 29-year-old male. His CDAI score increased from 62 to 278 and faecal calprotectin increased from 117 to > 400 μg/g. No medications were recorded at baseline or follow-up. In his exit interview, he referred to his stomach “going a bit funny but it not being a complete flare” at approximately one third of the way through the exercise programme. He thought that this “mini flare” was related to stress and not the exercise, and he was well enough to continue exercising, completing 33 sessions. Further review of his results show that a FC result done in the 6 months prior to entry to the trial was > 400 μg/g. At the time of entry to the trial he was on no medication having previously been on anti-TNF which was stopped due to antibody formation and clinical remission and his faecal calprotectin was being monitored. It seems likely that the in-trial flare occurred due to the progressive nature of his disease whilst on no treatment. He has since started on vedolizumab with a good response.

The MICT participant was a 37-year-old female. She was stable on 15 mg/week methotrexate at baseline but and had switched to 50 mg/day azathioprine by 3 months due to troubling hair loss which she perceived as a side effect of methotrexate. Within a few weeks of that switch she was suffering symptoms of a relapse and her faecal calprotectin was raised. She also developed anaemia. Over the course of the trial her CDAI increased from 38 to 181, and faecal calprotectin from 46 to > 400 μg/g. She completed 25 exercise sessions. Of the missed sessions, six were missed due to ill health (five of which due to virus/vomiting). In her exit interview, she referred to feeling tired at the end of the supervised period, and she put this down to anaemia, which she had only recently become aware of and received treatment for. It seems possible that her relapse was related to her switch in medication. She was eventually started on infliximab with a good response.

Four adverse events were also reported during the trial; all within the HIIT group. Three were rated as non-serious but exercise-related. One participant experienced a mild headache and dizziness after exercise on two separate occasions. After clinical review, these symptoms were deemed to be related to dehydration-induced migraine. The participant was re-informed about appropriate dietary and hydration habits in relation to the exercise sessions. After this, these symptoms no longer occurred. For the other adverse event, one participant vomited 5 min after the end of a session. This was likely due to the participant having eaten immediately before the session. The participant was re-informed about appropriate timing of meals in relation to the exercise sessions. The final adverse event was unrelated to the trial and non-serious; a participant became ill with a chest infection shortly after randomisation, resulting in them missing all of their exercise sessions.

## Discussion

A key finding of this pilot randomised controlled trial was that the pre-specified criteria for progressing to a full-scale trial of supervised exercise training in CD were all satisfied. The minimum recruitment target was achieved, and rates of exercise attendance and outcome completion were good. Interview feedback about the exercise programmes was generally positive, with most participants stating that they enjoyed attending and experienced fitness and health benefits. There were very few exercise-related adverse events.

This is the first study to test and demonstrate the feasibility and acceptability of HIIT in adults with CD. Several trials have shown HIIT to be a safe and effective exercise strategy in other clinical populations [13, 33, 34], but all previous prospective studies in CD have investigated low-to-moderate-intensity exercise programmes [7–11]. The reasons for this are unclear, but may include concerns that high-intensity exercise will acutely exacerbate inflammation and CD symptoms [4]. Such concerns are not supported by the current findings or other non-trial data. Indeed, both our cycle-based HIIT and MICT programmes had good attendance figures and positive feedback, with no participant reporting exercise causing a worsening of their symptoms. Interestingly, the majority of participants had a pre-randomisation preference for HIIT, suggesting that many patients want to exercise at a high-intensity and are not fearful of doing so (at least when under supervision and in a controlled environment). In previous work, Ploeger et al. [17] demonstrated that a single session of cycle-based HIIT was well-tolerated and did not exacerbate inflammation or disease symptoms in 15 teenagers with CD. Similarly, a conference abstract reporting a prospective study of seven adults with CD participating in high-intensity continuous exercise events such as triathlons, marathons, and long-distance bike races showed no abnormal elevation of faecal calprotectin measured at 24 h and one week after the event [35]. Five of the seven patients also had no change in their symptoms or disease activity scores. The two remaining patients showed elevated disease activity scores at 24 h after exercise, with scores returning to baseline within one week. Together, the available data appear promising regarding the safety of cycle-based HIIT; however, larger prospective studies are needed before firm recommendations can be made about the suitability of this type of training.

The pre-specified criteria for planning a full-scale trial were largely met. Sixty four percent of HIIT and MICT participants attended at least 24 of the 36 sessions (the aim was for at least 67%, this was achieved in MICT group but not in HIIT). A total of 36 participants were recruited over 17 months (mean 2.4 per month), with 27 randomised in the first 12 months. The 3-month response rate exceeded 67%. We therefore plan to progress to a full-scale trial, with some changes, that would have a main aim of determining the efficacy and safety of supervised exercise training in people with CD. The findings from our pilot work have implications for this future trial, and the proposed changes to study design are summarised in Additional file 3. The main changes relate to the intervention. Firstly, we plan to investigate one exercise programme instead of two (i.e., change to a 2-arm, exercise versus control design). Although this will remove the ability to compare different exercise programmes, it will simplify the design and make the recruitment target more attainable. Secondly, we plan to expand the exercise regime to include resistance and flexibility exercises. The addition of resistance training, the benefits of which we are currently investigating [36], will ensure the programme aligns with global recommendations on physical activity for health [37], and promote improvements in skeletal muscle function and bone strength [38], both of which are commonly impaired in people with CD [3, 39, 40]. For the aerobic component, we plan to use a mixture of HIIT and MICT because of evidence that doing so increases the likelihood of an improvement in cardiorespiratory fitness being observed [41], and that variety can support regular attendance [42]. Offering sessions on weekends might also improve attendance rates. This was not feasible in the current study because the university facilities were unavailable at weekends. For the future trial, we are exploring whether we could deliver the intervention in community-based exercise facilities that are open 7 days per week. Potential challenges include finding an exercise venue to pair with each of the hospital sites, and ensuring appropriate staffing.

Strengths of this study include blinded outcome assessment, low rates of attrition and missing data, and exercise sessions being consistently delivered as planned (when attended). The study did have some limitations, however. Firstly, the intentionally small sample size makes the study underpowered to assess efficacy, and the upper target sample size of 45 was not achieved. However, our preliminary data could be used in a meta-analysis in the future. A second limitation was the use of self-reported physical activity, which has been shown to be inaccurate when compared with objective measurement from devices such as accelerometers [43]. Thirdly, we did not use endoscopies to directly visualise the effect of exercise on the gastrointestinal tract. These two limitations can be addressed easily in the future trial by using accelerometry and capsule endoscopic evaluation, respectively. A fourth limitation was that participants were not blinded to group allocation during follow-up, making the patient-reported outcomes susceptible to bias [44]. Using a control condition that matches the exercise programme(s) for attention (e.g., flexibility or light resistance training) is a potential approach to minimising the risk of this bias. Finally, uncertainty remains about how successful recruitment and retention would be at other potential trial sites. Given that many more sites would be required in a subsequent trial, the continued monitoring of feasibility issues through an internal pilot phase would be beneficial, particularly within the first year of recruitment.

## Conclusion

In conclusion, cycle-based HIIT and MICT are feasible and acceptable exercise strategies in adults with quiescent or mildly-active CD. Larger-scale trials are needed to provide precise estimates of the benefits and harms of different exercise programmes, and our findings suggest that a multi-centre trial of supervised exercise training is feasible in the UK. Physical exercise remains a potentially useful adjunct therapy and lifestyle behaviour in CD.

## Additional files

Additional file 1: Summary of telephone interview data. (DOCX 14 kb)

Additional file 2: Summary of recruitment survey data. (DOCX 12 kb)

Additional file 3: Implications and proposed changes for the full-scale trial. (DOCX 16 kb)
