# Supplementary material for: High-intensity interval training and moderate-intensity continuous training in adults with Crohn’s disease: a pilot randomised controlled trial
Source: BMC Gastroenterol. 2019 Jan 29;19:19. doi: 10.1186/s12876-019-0936-x (PMC6352351; doi:10.1186/s12876-019-0936-x)
Supplement: Supplementary file 1 — Summary of telephone interview data. (DOCX 14 kb) [file 12876_2019_936_MOESM1_ESM.docx]

**Additional File 1 - Summary of telephone interview data**

Thirty-two (89%) participants completed a telephone exit interview. Of the four participants not interviewed, three could not be contacted, and one was not done because the participant missed all exercise and assessment sessions. A further one interview could not be analysed due to a poor quality of recording.

The participants had a mixed history of exercise. Some had previously undertaken regularly structured exercise such as gym training or running, whereas others had undertaken moderate-intensity physical activity as part of daily-life, e.g. walking or cycling. As per the eligibility criteria, no participants were completing >90 min/week of purposeful endurance-type exercise in the month before recruitment. No participants recalled receiving any specific advice or support regarding exercise as part of the management of their Crohn’s disease.

Twenty-six participants stated their mode of recruitment. The majority (15/26, 58%) were recruited via face-to-face approach in clinic; however, others were recruited after they responded to advertisements in the Crohn’s and Colitis UK magazine and website (n=7), a poster advertisement in clinic (n=2), or a letter of invitation sent via post (n=1), suggesting that using a range of recruitment strategies is beneficial. Interviewees offered the following suggestions about why some people might not enrol on a study like this: difficulty with travelling to study sites (n=4); lack of motivation (n=3); active Crohn’s or other limiting health conditions (n=3); lack of time (n=3); not liking exercise (n=1), and; concern that exercise may do more harm than good (n=1).

Participants cited various reasons for enrolling including potential health benefits (n=20), altruistic motives (n=12), and intellectual reasons (n=6; e.g. “never taken part in a study”). Some participants (n=10) also saw the study as a good way to kick-start an exercise regime.

Feedback on the study procedures was mostly positive and all participants said that they would recommend the study to other people with Crohn’s disease. All respondents felt well-informed prior to enrolment, and the combination of verbal and written information was valued. One participant would have liked more information on what the exercise test involved, and another on the location and content of the exercise sessions, but otherwise participants did not have any suggestions on how the information provided at the point of recruitment could have been improved. Regarding study assessments, one participant would have preferred to give blood at the hospital rather than the university because the hospital venepuncture staff are more experienced and have access to a wider range of sampling consumables. One participant thought that the questionnaires were too long, and another thought that some of the questions were difficult to answer. The exercise test was considered hard by all participants, but relevant and worthwhile doing.

The majority of participants had a pre-randomisation preference for being allocated to the HIIT group, with reasons including wanting to be challenged, and perceiving that it would be better for their fitness. The requirement for a control group was appreciated, and although control participants were disappointed with their allocation, they were happy to complete the study. Only one exercise participant said that they would have dropped out if they had been allocated to control.

Participants had mixed views about there being three sessions per week. Some (n=12) stated that this frequency was, or would have been, difficult to adhere to, whereas others felt this frequency to be achievable (n=11) and necessary for improving fitness (n=6). Two participants indicated that they would not have achieved this frequency had the session times not been as flexible. Three other participants stated that the frequency would have been more achievable had weekend sessions also been offered. One participant said that they needed a day off between sessions for recovery.

There was little feedback on the intensity of training; no one stated either training programme as being too hard or too easy. Two participants were initially concerned that the HIIT might be too hard, but found that this did not turn out to be the case.

One person commented on the programme duration (12 weeks) initially seeming long, but that they ended up wanting to continue for longer when reaching the end.

All participants found cycling to be an acceptable mode of exercise, with some recognising that it can be carefully controlled and is suitable for a range of fitness levels. However, two participants said that the seat was a bit uncomfortable. Six participants stated that they would have liked to also try other exercise modes including muscle-strengthening exercises (n=4), running (n=1), and arm-cranking (n=1). Another six participants stated that they were glad running was excluded, with two participants explaining that it has previously caused them to experience bowel urgency.

Most participants did not have a preference for where the exercise was conducted. Three participants preferred it being in a university rather than a hospital, but one participant would have preferred the hospital setting. One participant appreciated the privacy that the university setting gave her, but two others would have preferred to exercise with other people. The main other point that was raised about the setting was that, for some people (n=10), it was quite far to travel to.

Participants valued the exercise sessions being supervised, but no one felt that the sessions needed to medically-supervised (i.e. supervision by a non-medical exercise professional was acceptable). Two participants stated that the exercise sessions would have been boring had there not been someone there to talk to.

Participants valued receiving information about their progress (i.e. changes in fitness and health measures) and reports summarising both their individual results and the overall results of the study.

The interviewees reported a range of physical benefits from participating in the exercise programmes, including feeling fitter (n=8) and more energised (n=8), and having a thinner waist (n=1) and more-defined thigh muscles (n=2). Five participants also reported disease-specific benefits, such as reduced inflammation (n=1; based on routine colonoscopy findings), less frequent bowel movements (n=1), and a “calmer gut” (n=1). Mental benefits were less frequently cited, but included generally feeling better (n=2), and improvements in wellbeing (n=3) and mood (n=2). Eight participants said that the study had increased their motivation to exercise in the future, and 12 participants said that they had continued exercising (a variety of regimes) since finishing the supervised sessions. One control participant explained that they had started doing aerobic and strength training 3-4 times per week during the study.

Seventeen interviewees responded “yes” when asked if the NHS should offer supervised exercise training to people with Crohn’s disease. A further four interviewees responded “no”, but suggested that advice and education on exercise should be provided to patients. There was no consensus amongst participants about how much they would be willing to spend on exercise if supervised exercise was promoted but not freely available (range £3-30 per session and £10-100 per month).
