# Supplementary material for: High-intensity interval training and moderate-intensity continuous training in adults with Crohn’s disease: a pilot randomised controlled trial
Source: BMC Gastroenterol. 2019 Jan 29;19:19. doi: 10.1186/s12876-019-0936-x (PMC6352351; doi:10.1186/s12876-019-0936-x)
Supplement: Supplementary file 2 — Summary of recruitment survey data. (DOCX 12 kb) [file 12876_2019_936_MOESM2_ESM.docx]

**Additional File 2 - Summary of recruitment survey data**

Five researchers from across the three trial sites completed the survey of barriers and facilitators to recruitment. Two of the five researchers were site principal investigators, two were research nurses, and the other was a clinical trials assistant. The survey assessed recruitment experiences across six categories of factors: (i) trial, (ii) site, (iii) patient, (iv) clinical team, (v) information and consent and (vi) study team.

Factors that were generally rated as facilitators:

(i) Trial – publicity by the trial team; external publicity; trial management

(ii) Site – choice of recruitment setting; local research culture

(iii) Patient – patients’ attitudes towards exercise interventions

(iv) Clinical team – research experience of the team; motivation of clinical team; perceived importance of the particular research question; communication skills of clinical team; clinician attitude to involving patients in research

(v) Information and consent – clarity in presentation of trial information; time and setting of consent seeking; senior doctors and nurses seeking consent; experience and training of clinical team seeking consent

(vi) Study team – motivation of the study team at site; communication and coordination between study team members at site; communication and coordination between study team at site and the external trial management team; research experience of PI and study team members at site

Factors that were generally rated as barriers:

(i) Trial – none

(ii) Site – none

(iii) Patient - duration of trial and follow up; additional travel and extra costs

(iv) Clinical team – clinical workload; difficulty in approaching patients for consent

(v) Information and consent – none

(vi) Study team – none

Various strategies were implemented during the trial to try and enhance recruitment, which had varying success. Strategies included (i) providing potentially-eligible patients with stool samples pots ahead of their clinic visit so that they could bring a stool sample to the clinic and not have to make an additional visit (subjective assessment - useful); (ii) re-screening of patients that had failed initial screening due to raised FCP over study limit (useful); (iii) research nurses attending clinics and actively recruiting patients (useful); (iv) reminders to all clinicians at the beginning of clinics to consider patients for the trial (partly successful/useful); (v) posters in the waiting room about clinical research in IBD (useful); posting out invitation letters and information sheets to potentially-eligible patients on a local database (practical and theoretically useful, but only a couple of people responded via this method); (vi) regular team meetings (useful).

Ideas were offered about how the trial could have been organised differently to improve recruitment: (i) having a greater choice of sites for where the exercise is completed, including the recruiting sites (hospitals); (ii) having more recruiting sites; (iii) having more trial advertisements via online patient groups and charities; (iv) having a dedicated research assistant to identify and send out information sheets to all potential subjects.
