# Supplementary material for: High-intensity interval training and moderate-intensity continuous training in adults with Crohn’s disease: a pilot randomised controlled trial
Source: BMC Gastroenterol. 2019 Jan 29;19:19. doi: 10.1186/s12876-019-0936-x (PMC6352351; doi:10.1186/s12876-019-0936-x)
Supplement: Supplementary file 3 — Implications and proposed changes for the full-scale trial. (DOCX 16 kb) [file 12876_2019_936_MOESM3_ESM.docx]

**Additional File 3. Implications and proposed changes for the full-scale trial**

| **Methodological issue** | **Key findings or issues** | **Implications and proposed changes for the full-scale trial** |
| --- | --- | --- |
| Study set-up | There were no major problems in obtaining NHS Ethics or Research Management approvals; however slow processes in the Human Resources Department at the University of East London meant that we were delayed in being able to recruit a research assistant by about 4 months | Implication – Trial Management team to engage with HR departments about trial staff appointments as soon as possible during study set-up |
| Eligibility | The most common reason for exclusion was having active disease; site pre-screening forms were incomplete so rates of screening and eligibility could not be determined | Implication – include more robust processes and training for the collection of pre-screening data  Proposed change – remove autoimmune disease as an exclusion criterion because in hindsight this was unnecessary |
| Recruitment | By recruiting 36 participants, we achieved our minimum recruitment target (n≥24) but not our ideal target (n=45); some sites recruited better than others; most participants were recruited via face-to-face approach in clinic or advertisements posted by Crohn’s and Colitis UK | Implications – continue to use multiple recruitment strategies; include an internal pilot phase to provide ongoing monitoring of recruitment issues; only include sites that have a research nurse available to support recruitment  Proposed changes – extend the recruitment period and include more sites to accommodate the greater sample size |
| Randomisation procedures | The randomisation process worked well | Implication – include an internal pilot phase to provide ongoing monitoring of the randomisation procedures |
| Blinding of participants | Participants were not blinded to group allocation during follow-up | Potential change – use a control group that matches the exercise programme for attention (e.g., flexibility training) |
| Blinding of outcome assessors | Assessors of the anthropometric, cardiorespiratory fitness and disease activity outcome measures were successfully blinded to group allocation | Implication – costing of the full-scale trial should permit funding of blinded outcome assessment at all trial sites |
| Other aspects of outcome assessment | We used self-reported rather than objective measures of physical activity; we did not use endoscopies to directly visualise the effect of exercise on the gastrointestinal tract | Proposed changes – use tri-axial accelerometers to objectively measure physical activity; include endoscopic evaluation of Crohn’s disease activity  Potential additional change – include monetary incentives and recorded delivery to optimise questionnaire response rates |
| Adherence with the intervention | The overall attendance rate was good, but this could have been better as some participants struggled to fit their sessions around other commitments (e.g., work) | Implication – continue to offer flexibility with the timing of sessions; prioritise setting up sites that have training venues with good accessibility  Proposed change – include secondary analyses exploring the impact of non- and partial adherence on estimates of efficacy  Potential additional change – collaborate with an organisation that manages community-based exercise facilities where the intervention could be delivered 7 days per week |
| Acceptability of the intervention to participants | Interview feedback about the exercise programmes was generally positive; most participants (74%) had a pre-randomisation preference for high-intensity interval training (HIIT), although two participants were concerned that HIIT would be too hard; some participants would have liked greater variety in their training programme | Implications – reconsider how the exercise programme is presented to potential participants during the recruitment process (i.e. try and allay any concerns about difficulty); use an intervention that has more variety (see next row) |
| Other aspects of the intervention | We did not include muscle and bone strengthening and balance activities in the exercise programmes; testing two exercise programmes in a 3-arm trial will require many more participants than testing one programme in a 2-arm trial | Proposed changes – test a single exercise programme that includes a combination of aerobic, resistance, balance and flexibility exercises  Potential additional change – include a back-up option of home-based training if a participant is unable to attend a supervised session |
| Retention | No participants withdrew from the study and outcome completion rates were good (89-97%) | Implication – include an internal pilot phase to provide ongoing monitoring of retention issues |
| Logistics of multi-centre procedures | The multicentre procedures worked as planned, with good communication maintained between (i) the university and hospital pairings and (ii) the Trial Management Group and all sites | Proposed change – as the full-scale trial will involve many more sites, we propose that it would be necessary to collaborate with a Clinical Trials Unit, which would be responsible for coordinating the trial  Potential change – as eluded above, we may pursue delivering the intervention via a community exercise provider rather than in university exercise science facilities |
